# The Fourth International Neonatal and Maternal Immunization Symposium (INMIS 2017): Toward Integrating Maternal and Infant Immunization Programs

**DOI:** 10.1128/mSphere.00221-18

**Published:** 2018-11-07

**Authors:** Flor M. Munoz, Pierre Van Damme, Ener Dinleyici, Ed Clarke, Beate Kampmann, Paul T. Heath, Ofer Levy, Elke Leuridan, Clare Cutland, Ajoke Sobanjo-ter Meulen, Arnaud Marchant

**Affiliations:** a Department of Pediatrics, Baylor College of Medicine, Houston, Texas, USA; b Centre for the Evaluation of Vaccination, Vaccine & Infectious Disease Institute, University of Antwerp, Antwerp, Belgium; c Eskisehir Osmangazi University Faculty of Medicine, Eskisehir, Turkey; d Medical Research Council Unit, The Gambia at the London School of Hygiene and Tropical Medicine (MRCG at LSHTM), Banjul, The Gambia; e Imperial College of London, London, United Kingdom; f St. George’s University of London, London, United Kingdom; g Precision Vaccines Program, Division of Infectious Diseases, Boston Children’s Hospital, Boston, Massachusetts, USA; h Harvard Medical School, Boston, Massachusetts, USA; i DST/NRF Respiratory and Meningeal Pathogens Research Unit, Wits Health Consortium, Faculty of Health Sciences, University of the Witwatersrand, Johannesburg, South Africa; j The Bill & Melinda Gates Foundation, Seattle, Washington, USA; k Institute for Medical Immunology, Universite Libre de Bruxelles, Brussels, Belgium; l Department of Molecular Virology and Microbiology, Baylor College of Medicine, Houston, Texas, USA; UMKC School of Medicine

**Keywords:** maternal immunization, neonatal, infant, vaccines in pregnancy, vaccination, immunology, integration, implementation

## Abstract

Prevention of serious infections in pregnant mothers, newborns, and young infants through immunization during pregnancy and in early life has the potential to further reduce maternal and neonatal morbidity and mortality worldwide. In the past decade, research in this field has advanced substantially, from the understanding of the biology and immunology of pregnancy and early life, to the active development of several candidate vaccines, for which challenges and opportunities for global implementation are under consideration. Experts from academia, industry, regulatory and funding agencies, public health, and international organizations met in Brussels (Belgium) from 10 to 12 September 2017, at the 4th International Neonatal and Maternal Immunization Symposium (INMIS), to review the most relevant advances in maternal and neonatal immunization. The overarching focus of the conference was to identify the path forward to achieve integration of maternal and early life immunization strategies for the successful implementation of vaccines in antenatal care and pediatric programs for reduction of maternal and infant mortality worldwide.

**IMPORTANCE** This report provides an overview of the proceedings of the 4th International Maternal and Neonatal Immunization Symposium, where presentations focused on the state-of-the-art research on the development and implementation of vaccines given during pregnancy for the protection of mothers and infants.

## Abstract (precis)

This report provides an overview of the proceedings of the 4th International Maternal and Neonatal Immunization Symposium, where presentations focused on the state-of-the-art research on the development and implementation of vaccines given during pregnancy for the protection of mothers and infants.

## INTRODUCTION

### The integration of infant and maternal immunization: a global challenge.

Global maternal and infant mortality remains unacceptably high in the 21st century. Despite a 44% reduction since 1990, the global maternal mortality ratio was estimated to be 216 (207 to 249) per 100,000 live births in 2015, with 303,000 (291,000 to 349,000) maternal deaths (830 per day), mostly associated with pregnancy and childbirth complications, 99% occurring in the developing world (1). Similarly, 4.9 million children under 5 years of age died in 2015 (nearly 16,000 every day), with neonatal mortality accounting for approximately 45% of all deaths (2). The World Health Organization has therefore included in their 2030 Sustainable Development Goal (SDG) targets the goal to reduce the global maternal mortality ratio to <70 per 100,000 live births and end preventable deaths of newborns and children under 5 years of age, aiming for all countries to reduce neonatal mortality to ≤12 per 1,000 live births and under-5 mortality to ≤25 per 1,000 live births (2). To achieve these targets, interventions addressing the prominent causes of mortality in these target groups are required. Expansion, enhancement, and integration of maternal and early life immunization, key public health interventions, have the potential to address some of these goals (3). Pregnant women and infants have a high risk of exposure to infectious disease, and vaccines administered during pregnancy and/or early life offer the potential to protect, depending on the vaccine, mothers against infection-related morbidity and mortality and infants against infection-related morbidity, neonatal death, stillbirth, and preterm birth. The feasibility of maternal immunization is supported by the success of the longstanding Maternal Neonatal Tetanus Elimination (MNTE) program. Given the growing number of vaccines recommended for use during pregnancy (i.e., influenza and pertussis), and of new maternal vaccines in development (i.e., respiratory syncytial virus [RSV] and group B streptococcus [GBS]), it is critical to understand the challenges and opportunities to implement maternal immunization in areas where vulnerable populations would benefit the most, including low- and middle-income countries (LMIC), leveraging existing antenatal care (ANC) programs. In 2015, experts at a Bill & Melinda Gates Foundation (BMGF)-convened meeting on maternal immunization in resource-limited settings agreed that maternal immunization must be integrated within the ANC platform (4). This approach is supported by the Strategic Advisory Group of Experts on Immunization (SAGE), who have advised the World Health Organization (WHO) to generate generalizable data on the best ways to integrate maternal immunization into routine antenatal care in low-resource settings. Several global projects are addressing this mandate and identifying opportunities and challenges for the introduction of new vaccines for maternal immunization: the Maternal Influenza Immunization project, which provides guidelines for the implementation of influenza vaccination in pregnant women in LMIC, and the Maternal Immunization in Antenatal Care Situational Analysis (MIACSA), which aims to evaluate tetanus maternal vaccine delivery via existing ANC programs and identify opportunities and challenges for the introduction of new vaccines for maternal immunization (5). Vaccines specifically being developed for maternal immunization include a vaccine against GBS, the leading etiologic agent of neonatal sepsis and neonatal meningitis plus substantial maternal morbidity/mortality during pregnancy and postpartum, and an RSV vaccine in order to prevent the substantial morbidity and mortality caused by RSV in young infants. Similarly, PATH’s Advancing Maternal Immunization (AMI) collaboration, in collaboration with WHO, evaluates RSV implementation as a maternal vaccine, particularly in LMIC (https://sites.path.org/cvia/our-disease-targets/respiratory-syncytial-virus/advancing-maternal-immunization-ami/).

During 2015 and 2016, the WHO Product Development for Vaccines Advisory Committee (PDVAC) identified as a priority the development of GBS vaccines suitable for maternal immunization in pregnancy and use in LMIC. In response, WHO developed a GBS Preferred Product Characteristics (PPC) guideline and a Vaccine Development Technology Roadmap to focus on activities leading to development, testing, licensure, and global availability of GBS vaccines. These documents are available at http://www.who.int/immunization/research/development/ppc_groupb_strepvaccines/en/. In April 2016, SAGE acknowledged the high importance of RSV vaccine development to prevent pediatric morbidity and mortality. Since then, an RSV vaccine has been advanced to phase 3 clinical trials in pregnant women. To further inform vaccine development, licensure, and prequalification, WHO has developed guidance on RSV vaccine efficacy trial design (prevention of acute disease) with focus on LMIC, an RSV vaccine technology roadmap, and an RSV vaccine Preferred Product Characteristics (PPC) document to guide vaccine development and evaluation. These can be found at http://www.who.int/immunization/research/development/ppc_rsv_vaccines/en/. Regulatory and physical standards for RSV vaccines, appraisal of data relevant to RSV vaccination policies, and vaccine use and guidance on long-term vaccine evaluation are included in these documents.

The 4th International Neonatal and Maternal Immunization Symposium (INMIS) convened experts from academia, industry, regulatory and funding agencies, public health, and international organizations in Brussels (Belgium) from 10 to 12 September 2017, to review the most relevant advances in maternal and neonatal immunization. The overarching focus of the conference was to identify the path forward to achieve integration of maternal and early life immunization strategies for the successful implementation of vaccines in antenatal care and the harmonization of vaccination and maternal and neonatal health care programs to impact maternal and infant mortality worldwide. Nearly 300 participants attended the two-and-a-half-day meeting that included 16 invited expert presentations, 25 oral presentations from submitted abstracts, 30 poster presentations, and 2 expert panel discussions. The meeting sessions were organized to begin on day 1 with the general topic of immunology, including understanding antibodies and their transfer from mother to infant, neonatal immunization, and heterologous immunity. The second day was dedicated to providing updates on vaccines under development for maternal and neonatal immunization, addressing also safety, awareness, and perception issues. Finally, the third day focused on implementation aspects of maternal and neonatal immunization worldwide and future directions, including new and future targets of maternal and neonatal immunization.

### Immunology.

**(i) Antibodies and their transfer.** As an introduction to the development of new vaccines, Margaret Ackerman (Dartmouth College, USA) discussed how vaccine design can be informed by systems serology, an approach she pursues in collaboration with Galit Alter (Ragon Institute, Cambridge, MA, USA). Using HIV infection and HIV vaccines as examples, Dr. Ackerman illustrated how machine-learning tools can be employed to learn the principles of an effective antibody (Ab) response in human subjects. Specificity and neutralizing activity of antibodies (Abs) are dependent on their Fab (fragment antigen binding) domain, whereas the Fc (fragment crystallizing) domain mediates effector functions through their interactions with innate immune cells and complement. The importance of these interactions is illustrated by the reduced efficacy of HIV broadly neutralizing antibodies with mutations preventing their interaction with Fc receptors (6). The effector functions that can be mediated by IgG Fc domain are diversified by the different IgG subclasses and by posttranslational modifications primarily involving glycosylation. The importance of Fc glycosylation is illustrated by the impact of maternal antiplatelet IgG fucosylation on the severity of fetal thrombocytopenia. To gain insight into the rules governing vaccine efficacy, Ab responses to two HIV vaccine formulations based on the same antigens (VAX003 versus Rv144) were compared. A large range of features was assessed to broadly characterize Abs and identify correlates of protection. Structural Ab features could be linked to receptor binding and functional properties, and these could be correlated with vaccine efficacy. These observations support the notion that systems serology could inform vaccine design for diverse populations, including pregnant women and infants.

Dana Wolf (Hadassah University Hospital, Israel) discussed how congenital infections provide insight in placental biology and transfer of maternal immunity. The placenta plays a central role in maternal tolerance of the fetus and actively transports maternal IgG via the neonatal Fc receptor expressed by syncytiotrophoblasts. The placenta is also an innate immune barrier against pathogens. Syncytiotrophoblasts are known to be resistant to infection with multiple pathogens. It is thought that fetal infection may involve the transplacental transport of complexes formed by pathogens and maternal antibodies. On the other hand, infection of the decidua, forming the maternal component of the placenta, is likely to be the initiation of pathogen transmission to the fetus. Dr Wolf presented the development of *ex vivo* models of infection of decidua cultures allowing the characterization of pathogen spread and innate immune responses. These models demonstrated that cytomegalovirus (CMV) spreads from cell to cell whereas Zika virus (ZIKV) spreads via the extracellular space. Gene expression analyses revealed that CMV and ZIKV induce distinct placental responses that may underlie distinct mechanisms of viral control and tissue damage. In-depth analysis of gene expression indicated that the intrinsic cellular restriction factor APOBEC3A (A3A) may be an important innate mechanism of CMV control at the placental level (7). This work should help the development of interventions protecting placental functions and preventing pathogen transmission following maternal infections.

Thao Mai Phuong Tran (University of Hasselt, Belgium) discussed the mathematical modeling of maternal antibody titers in infants, after maternal immunization in Belgium and Vietnam with different brands of pertussis-containing vaccines. Different maternal antibody kinetics but similar half-lives were noted in young infants in both countries (concentrations at birth were lower in infants in Vietnam than in infants in Belgium). Future research will assess the impact of the timing of maternal vaccination at different stages of gestation on antibody concentrations in infants at birth, to further refine these models. Mustapha Jaiteh (Medical Research Council Unit, The Gambia [MRCG], at the London School of Tropical Medicine [LSHTM]) presented preliminary results suggesting an impact of maternal vaccination with 13-valent pneumococcal conjugate vaccine on the presence of vaccine antigen-specific memory B cells in colostrum and a potential mechanism underlying neonatal protection via maternal immunization through breast milk antibodies.

**(ii) Neonatal immunization.** Despite challenges inherent to vaccine development in the very young, there is a robust rationale for continued vaccine development for this population, including (a) birth is the most reliable point of health care contact and therefore neonatal vaccines achieve high population penetration; (b) neonatal immunization is a logical approach to protect preterm infants, who represent ∼11% of all live births and are often born prior to transfer of maternal antibodies (MatAbs) (such that maternal immunization may not be protective); and (c) growing evidence that the heterologous benefits of certain vaccines such as BCG may be greatest in early life (8).

Beate Kampmann (Imperial College London and MRCG at LSHTM, The Gambia) challenged the view that newborns do not respond well to vaccines and emphasized the importance of considering differences between newborns and between vaccines. Immune ontogeny in early life shapes innate and adaptive responses and is influenced by maternal cofactors (9). Immune plasticity is required to integrate genetic and environmental factors and to adapt to the *ex utero* environment. Immune components central to vaccine responses, including antigen-presenting cells, B cells, and T cells, function differently at birth than later in life. Maternal cofactors influencing immune ontogeny and immune responses in early life include chronic maternal infections, nutrition, and the microbiome and the levels and specificity of maternally acquired antibodies. Vaccines currently given at birth provide strong evidence that protective immunity can be induced by vaccination and can also inform on the potential of neonates to develop specific immune responses and on the impact of cofactors. Studies of BCG immunization have demonstrated that Th1 responses can be induced at birth and that BCG vaccination can have heterologous effects on the response to other vaccines and on overall infant mortality (8, 10). Studies of hepatitis B and oral polio immunization at birth indicated that priming of memory B cells can be achieved in early life, allowing the option to immunize infants at birth. An ideal vaccine to be administered to neonates would induce rapid immune response even in the presence of MatAbs and would have an optimal safety profile (11).

Nicholas Wood (University of Sydney, Australia) discussed the role of neonatal immunization for protection against bacterial pathogens, including pertussis, Haemophilus influenzae type b (Hib), and pneumococcus. The majority of deaths due to whooping cough occur in the first two months of life. Pertussis vaccination in early life has resulted in variable outcomes. Historical studies suggested that immune tolerance may result from neonatal pertussis immunization with whole-cell vaccines. More recent studies showed that acellular pertussis vaccination at birth increases the response to the second dose of vaccine at 2 months of life (a priming role). Some studies showed reduced responses to vaccines concomitantly administered with the second pertussis vaccine dose. Another limitation of pertussis immunization at birth may be the induction of Th2-polarized cellular immune responses. Neonatal pertussis immunization may be indicated in babies born to mothers who have low Ab levels, reduced response to pertussis vaccine, or reduced transfer of MatAbs. From a public health point of view, the feasibility of such indications can be questioned. Several studies of neonatal Hib immunization showed good safety and priming of booster responses with polyribosylribitol phosphate-diphtheria CRM 197 protein (PRP-CRM) and PRP-tetanus protein (PRP-T) vaccines but reduced booster responses to a PRP-outer membrane protein (PRP-OMP) vaccine. Studies of neonatal immunization with pneumococcal conjugate vaccine showed good safety and early induction of Abs, but their impact on invasive disease or carriage has not yet been demonstrated.

Merryn Voysey (University of Oxford, United Kingdom) discussed prevalence and decay of MatAbs from different pneumococcal and meningococcal vaccine trials and showed differences between serotypes, serogroups, and countries (12). The presence of MatAbs results in interference with active infant immunization. These data are useful for modeling the impact of programs that utilize a combination of maternal and infant vaccination strategies. Ofer Levy (Precision Vaccines Program, Boston Children’s Hospital and Harvard Medical School, USA) presented preliminary *in vitro* studies employing human whole-blood assays raising the possibility that BCG formulation heterogeneity may contribute to variability in clinical benefit, including “off-target” heterologous protection, highlighting the need to further standardize and compare different BCG vaccines to optimize benefit. Data presented by Ed Clarke (MRCG at LSHTM, The Gambia) showed that vaccine-specific memory B cell responses to pneumococcus antigens can be detected in mothers and infants after maternal immunization with 13-valent pneumococcal vaccine. Gaelle Noel (University of Maryland School of Medicine, USA) presented data demonstrating that breast milk enhances the intestinal mucosal barrier in a pediatric human enteroid model. John Ojal’s (KEMRI-Wellcome Trust Research Program) data indicated that naturally acquired MatAbs against pneumococcal proteins protect infants from carriage whereas vaccine-induced antipolysaccharide Abs may require concentrations higher than those induced by natural immunity to be effective (13).

**(iii) Heterologous immunity.** Mihai Netea (Radboud University, The Netherlands) reviewed the concept of innate immune memory and heterologous effects of vaccines. He highlighted that upon introduction of BCG vaccine in Sweden in 1927 to 1931, infant mortality dropped from 11% to 4% in a manner that could not be accounted for solely by prevention of tuberculosis. Subsequently, randomized studies of low-birth-weight newborns in Guinea Bissau demonstrated that newborns who received BCG at birth versus at 2 months had an ∼50% reduction of mortality. Several investigators have hypothesized that the mechanisms underlying such rapid BCG effects may relate to innate immunity. From an evolutionary perspective, it may not seem logical that vertebrates, representing only 5% of all known organisms, would rely on only adaptive responses for immunological memory. Moreover, broad systemic acquired resistance to pathogens is well described in plants and can be transmitted to the next generations via epigenetic mechanisms. Such innate memory also exists in mammals but has been less well studied. For example, in mice lacking T and B lymphocytes, myeloid cells provide protection following vaccination with *Candida*. This protection also involves epigenetic mechanisms. Key histone modifications increasing access to chromatin are detected in primed monocytes, and blocking these modifications inhibits cell priming. These epigenetic modifications are related to metabolic changes, including changes in the Krebs cycle, also demonstrable in human adults following BCG vaccination. Their relevance for antimicrobial defense is supported by recent data indicating decreased viral replication in subjects who received BCG before yellow fever vaccination (14). Overall, it is evident that innate immune responses can change in an adaptive manner after infection or vaccination.

Carlo Pietrasanta (Boston Children’s Hospital and Harvard Medical School, USA) reported that certain combinations of Stimulator of Interferon Genes (STING) and Toll-like receptor (TLR) agonists act in synergy to activate Th1-polarizing responses from human neonatal antigen-presenting cells, suggesting that STING agonists, alone or combined with alum and/or TLR agonists, may be candidate adjuvants for early life immunization (15). Selena Alonso (Barcelona Institute for Global Health, Spain) discussed the impact of maternal infections on infant responses to vaccines. She presented results of a study conducted in Mozambique indicating altered immune responses to vaccine antigens (Hib, rotavirus, pertussis, and diphtheria) in infants born to mothers infected with HIV or malaria. Unexpectedly, intrapartum prophylaxis with mefloquine had a negative impact on vaccine responses. Jishnu Das (Ragon Institute, USA) described a study conducted in Belgium and showing that HIV-exposed uninfected infants are at higher risk of hospitalization for infection of various etiologies than unexposed infants. Risk of hospitalization was related to time at initiation of antiretroviral therapy in the pregnant women and was predicted by immune alterations measured at birth. The risk for hospitalization was 4-fold higher when mothers initiated therapy during pregnancy but not significantly increased when treatment was started before pregnancy (16).

### Update on vaccines under development for maternal and neonatal immunization.

**(i) RSV immunization in pregnancy.** Fiona Culley (Imperial College London, United Kingdom) reminded the audience that RSV is the leading single cause of hospitalization of infants worldwide and a significant cause of global deaths, especially in LMIC. As adults can be repeatedly infected with the same RSV strain, natural immunity may be only partially effective. Natural serum Ab is associated with protection, although nasal RSV-specific IgA may provide better correlation. The best evidence of protection comes from the success of monoclonal antibody (MAb) to F protein. On the other hand, the age profile of RSV disease supports an association between susceptibility and levels of MatAb. One modeling study showed that for every doubling of Ab by a vaccine, severe disease may be reduced by 30% and protection prolonged for an additional 50 days. Maternal immunization is an attractive option, and a number of vaccine candidates are under development and evaluation (15, 17). Factors that are important to consider for candidate vaccines include how much Ab is generated in the mother, the variability of the response, the best gestational age to vaccinate, the protection of the mother, the placental transfer of Ab, the impact of breast feeding, the persistence of the presence of passively acquired Abs, the possible interference with infant response to natural infection, and the potential effect of delaying the age of infection to later in childhood. Additional strategies to be considered include cocooning, the use of pediatric RSV vaccines, and of extended-half-life MAb formulations.

**(ii) GBS immunization in pregnancy.** Group B streptococcus is a leading cause of maternal and neonatal morbidity worldwide. Recent estimates associate GBS with at least 409,000 (uncertainty range [UR], 144,000 to 573,000) maternal-fetal-infant cases of disease and 147,000 (UR, 47,000 to 273,000) stillbirths and infant deaths annually. It is anticipated that an effective vaccine could substantially reduce the burden of GBS disease (18).

Shabir Madhi (University of Witwatersrand, South Africa) emphasized that GBS disease is underrecognized as a global cause of deaths and disability, especially in LMIC, in part because the current tools for measurement are suboptimal (19). Previous estimates show the virtual absence of GBS in Southeast Asia. This observation contrasts with the substantial maternal colonization rates in that setting. Underdetection of invasive infections may be due to the fact that 90% of early-onset disease occurs on day 1 of life and the bulk of disease and attributable infant mortality will be unrecognized if cases die at home or are not investigated adequately in health care facilities due to resource constraints. Even in high-income countries with effective use of intrapartum antibiotics, there remains a residual burden of early-onset disease, and intrapartum prophylaxis has had no impact on late-onset disease. Since the historical observation that lower levels of anticapsular Ab are correlated with invasive disease, work to define a threshold associated with protection has progressed but has been limited by the use of different and unstandardized assays. Recent studies of conjugate vaccines have demonstrated safety and immunogenicity with good persistence of Abs in vaccinated mothers, with variation by preexisting natural immunity, comorbidities, and ethnicity. Good placental transfer and Ab persistence in infants have also been observed, but long-term persistence of MatAb may be less important for GBS than for other infections such as RSV because the highest burden of GBS mortality is in the first week of life, and coverage for the first 3 months of life through maternal vaccination is sufficient to address both early- and late-onset disease. HIV infection is an important risk factor for infant GBS disease, and lower responses to candidate GBS vaccines have been seen in HIV-infected than in uninfected women. Dr. Madhi referred to two recent WHO publications describing the pathway for development and preferred product characteristics for GBS vaccines (20, 21).

Manu Chaudhary (Baylor College of Medicine, USA) shared the results of a study conducted in the United States where GBS colonization prevalence in women from India or of Indian descent living in the United States was comparable to that generally in the United States but higher than in India. Further, GBS colonization prevalence was higher in women of Indian descent who were born in the United States than in those born in India, while isolated GBS types were reflective of both countries, including type VII (prevalent in South Asia) and type IV, which is emerging in the United States (22).

**(iii) Pertussis immunization in pregnancy.** Kathryn Edwards (Vanderbilt University School of Medicine, USA) gave an overview on the historical perspective, the rationale, maternal-fetal antibody transfer, and the effectiveness and safety of maternal immunization against pertussis and discussed the impact of maternal antibody (MatAb) on infant immune responses to pertussis-containing vaccines. Evidence is robust that maternal immunization with acellular pertussis vaccines is safe and effective in protecting neonates and young infants from disease. This is in line with the increasing body of evidence supporting safety, immunogenicity, and protective efficacy of Tdap in pregnancy (23, 24). Studies evaluating the optimal timing of vaccination during pregnancy indicate that vaccination in the second or early third trimester might result in higher neonatal Ab concentrations (25, 26). The impact of MatAbs modifying the infant immune responses, however, is one of the remaining challenges of Tdap vaccination in pregnancy research (27–29). Finally, there is a theoretical concern about interference of CRM-conjugated vaccines with responses to infant vaccines—which has not been shown in these recent studies.

Nasamon Wanlapakorn (Chulalongkorn University, Thailand) presented results from an ongoing study in Thailand (2016 to 2018) where infants born to Tdap-vaccinated women are randomized to either whole-cell pertussis- or acellular pertussis-containing infant vaccines. The optimal timing of Tdap vaccination in pregnancy was calculated to be at least 8 weeks prior to delivery; the ratio of maternal/cord vaccine-induced Abs was higher when Tdap vaccine was offered earlier in pregnancy. Results on infant responses were not yet available.

Thomas Rice (Imperial College London, United Kingdom) reported on the MatImms study at Imperial College in the United Kingdom, which investigates the contributions of emerging pertussis strains to early cellular immune responses to vaccines, using a whole-blood stimulation assay (30). Elevated cellular immune responses in vaccinated women suggest that vaccination in pregnancy also boosts cellular responses in women. Strain-dependent responses were evident, and significantly higher Ab titers were present in the vaccinated women and persisted in their offspring until infant vaccination started. After 3 aP-containing infant vaccines, cytokine responses were again strain dependent and differed in some cytokines in infants.

Carolina Argondizo (Adolfo Lutz Institute, Brazil) also reported on the cellular immune response patterns of mothers and infants, using flow cytometry. Tdap in pregnancy enhanced anti-PT cellular immune responses in women, confirming boosting of cellular immunity at adult age. After 3 doses of wP-containing vaccines given to the infants, infant cellular immune responses were no different between the study groups born to vaccinated and to unvaccinated women, despite a blunting effect of MatAbs in infants born to vaccinated women.

In the discussion, the question was raised whether the above-mentioned studies can capture vaccine “nonspecific” or heterologous effects of maternal immunization, requiring the assessment of nonspecific features in the study design as well as larger study populations for broader documentation of disease morbidity and mortality. In summary, after a thorough review of the existing evidence on maternal Tdap vaccination benefits and remaining knowledge gaps, study results from Thailand, Brazil, and the United Kingdom could answer one of the remaining questions on the impact of maternal vaccination on cellular immune responses in both the women and their infants.

**Safety, awareness, and perception of maternal and neonatal immunization.** The next session was dedicated to examine perceptions related to maternal immunization globally and how they might impact vaccine uptake and the global efforts under way to strengthen and harmonize safety monitoring in this area.

**(i) Perception of risk related to maternal and neonatal immunization: the importance of building trust.** Heidi Larson (London School of Hygiene and Tropical Medicine, United Kingdom) opened the session and provided her insights into the issues related to vaccine acceptance. She highlighted the universally sensitive nature of pregnancy worldwide, irrespective of culture, and the fact that anxieties related to many vaccines have focused on their perceived effects on fertility and reproduction. HPV vaccines are a particular example. The fact that a health belief model incorporating only an individual’s understanding of the risks of the disease and the acceptability of the intervention represented an oversimplification and that the “emotional determinants of health” also needed to be included was also discussed. Dr. Larson highlighted that trust in product (i.e., the vaccine), provider, and policy or policy maker was required for effective vaccine delivery and that uptake was likely to be compromised if any such component was lacking. She also drew attention to the fact that vaccines targeting disease in newborns (e.g., pertussis) might be accepted differently from those predominantly targeting maternal disease (e.g., influenza). Finally, Dr. Larson described the efforts of the Vaccine Confidence Project (http://www.vaccineconfidence.org/) to establish estimates regarding population views of the importance, safety, effectiveness, and compatibility with religious beliefs and outlined the many local contextual factors influencing such estimates.

**(ii) Assessment of safety.** Linda Eckert (University of Washington, Seattle, WA, USA) described efforts to harmonize the assessment of the safety of vaccines in pregnancy, providing her perspectives as a front-line obstetrician. She first highlighted that safety represents a key obstacle to maternal immunization and that the efficient use of data to study outcomes requires agreement regarding the terms and definitions being used. Dr. Eckert described the Global Alignment of Immunization Safety Assessment in Pregnancy, the GAIA consortium based on the Brighton Collaboration, which has recently published 21 definitions (10 obstetric and 11 neonatal) for use in safety reporting after maternal immunization (http://gaia-consortium.net/). The CROWN initiative, which was set up by journal editors to establish a core outcome set for reporting obstetric and neonatal studies, was also described (http://www.crown-initiative.org/). A set of preterm birth core outcomes has recently been published by this group, and further outcomes are to follow. A number of other ongoing initiatives were described before Dr. Eckert concluded that we are poised for a new era in harmonization of safety reporting in the field of maternal and neonatal vaccination.

Clare Cutland (University of the Witwatersrand, South Africa) provided a perspective from LMIC on assessing the safety and efficacy of maternal vaccines, considering immunization data collected routinely in health care facilities in South Africa, The Gambia, Ghana, India, and Nepal. She highlighted that the collection of safety data in clinical trials and routinely following implementation would present different challenges. Paper-based, patient-held immunization records currently in use in these countries do not have fields for recoding of essential data, including vaccine lot number, manufacturer, volume, and site of immunization. A lack of reliable electricity supply and computer/tablet hardware availability in many LMIC limits electronic data capturing. In a clinical setting in South Africa, creative solutions such as the use of different sizes of local coins to allow evaluators to consistently measure the size of local vaccine reactions have been utilized. In the majority of LMIC, pregnancy records are maintained on paper cards, which are taken home by the mother postdelivery. No system for the central storage of antenatal data is in place, hampering assessment of background rates of maternal, fetal, and newborn adverse events and making retrospective studies nearly impossible. A number of steps that could be taken to enhance safety reporting in LMIC were described to ensure that the future introduction of vaccines where they are most needed will not be delayed.

Ousseny Zerbo (Kaiser Permanente Vaccine Study Center, USA) presented data generated in the Kaiser Permanente site in northern California, demonstrating the ability to conduct powerful analyses using extensive data on >145,000 deliveries. The study confirmed an absence of association between maternal influenza vaccination and different adverse birth outcomes, including preterm birth, low birth weight, and large and small sizes for gestational age (31).

Penda Johm (MRCG at LSHTM, The Gambia) discussed qualitative data generated in the context of a maternal Prevenar13 vaccination trial in The Gambia. She reported that both maternal and neonatal doses of the vaccine were perceived to be safe by study participants, although it was apparent that there was a variable level of understanding regarding the illnesses that the vaccine may prevent. Potential barriers to future uptake were identified and are the focus of further studies to be undertaken in The Gambia and Senegal, outside the sites already undertaking vaccine trials, in order to eliminate bias.

Steve Anderson (Food and Drug Administration, United States) described the work of the U.S. Food and Drug Administration (FDA) to develop capabilities to monitor the safety of vaccine use during pregnancy through the sentinel Initiative of the Post-licensure Rapid Immunization Safety Monitoring (PRISM) program (https://blogs.fda.gov/fdavoice/index.php/tag/sentinel-initiative/), an active surveillance system that analyzes information in electronic health care databases.

### Implementing maternal and neonatal immunization worldwide.

Philip Lambach (WHO Initiative for Vaccine Research) provided a global perspective on the opportunities and challenges for maternal immunization with a particular focus on the lessons learned from influenza vaccine introduction. He highlighted the gap in implementation research, the need to optimize delivery strategies for vaccines in pregnancy, and the importance placed on supporting such research as highlighted by WHO’s Strategic Advisory Group of Experts (SAGE) in 2015. The progress, challenges, and opportunities for maternal influenza immunization as identified by each of the WHO Regions were then described. While in the African region (AFRO) competing infectious disease priorities were of significant concern, low population uptake in the European region (EURO) was a particular challenge. Currently, the Panamerican Health Organization (PAHO) is reported as the region making the strongest progress overall. Dr. Lambach described the framework for introducing a new maternal vaccine into a country program starting from the decision by policy makers and moving through the role of those undertaking the implementation in planning the introduction, undertaking training and ensuring effective communication, and subsequently monitoring and evaluating the program. Finally, the “WHO Maternal Influenza Introduction Implementation Manual” was fittingly launched on the day of the meeting (12 September 2017) and was recommended to the audience as a source for Extended Program on Immunizations (EPI) managers and other interested parties planning such introductions (5). (Guidance documents can be found at http://www.who.int/immunization/research/development/influenza_maternal_immunization/en/index1.html.)

Edwin Asturias (University of Colorado, Denver, CO, USA) discussed the fact that the integration of maternal and young infant immunization programs must address the effects of both schedules on the safety, the immunogenicity, and the overall efficacy of the vaccines, as well as programmatic issues. An ideal schedule was considered to offer the best opportunity to provide optimal protection with the minimum of risk. It was highlighted that the 6-, 10-, and 14-week primary immunization schedule had been adopted to maximize vaccine coverage in early infancy as rapidly as possible and had been highly effective at preventing disease. However, in reality most infants receive their vaccines at an average of 2.5, 4.5, and 8 months of age. The many factors affecting an infant’s response to their primary series were listed, and Dr. Asturias reviewed a number of the key publications demonstrating the effects of MatAbs, including breast milk Abs, on infant vaccine immunogenicity (32–39). He also highlighted the growing appreciation of the role of the microbiota in infant immunity (40, 41). He concluded that true integration of maternal and infant immunization schedules might involve a delay in the administration of those vaccine antigens against which the infant was effectively protected by MatAbs until such Abs had waned sufficiently. This would also provide greater opportunities for the later booster vaccinations required for long-term protection and, in many cases, indirect protection in the population.

Begoña Martinez de Tejada (University Hospitals of Geneva, Switzerland) presented the results of two prospective, observational, nonrandomized studies comparing the transfer of antipertussis Abs to the newborn following vaccination in the second compared to the third trimester of pregnancy (25, 42). In both term and preterm infants, second-trimester vaccination conferred higher Ab concentrations to the newborn, and hence, a lower number of infants remained seronegative following second-trimester vaccination. Dr. Martinez de Tejada concluded with a recommendation for second-trimester pertussis vaccination.

Gayatri Amirthalingam (Public Health England, United Kingdom) discussed the successes and challenges of the maternal pertussis immunization program in England. The program was introduced in 2012 in response to a sharp increase in pertussis disease cases in those too young to have been vaccinated (23). Although the program was successfully implemented early on, coverage was increased when the start of the window for vaccination was broadened from >28 to >16 weeks of gestation, based on data from recent studies (as described by previous presenters). Over 90% vaccine effectiveness has been demonstrated consistently since introduction (23). In the United Kingdom, infant mortality from pertussis has substantially decreased since the introduction of maternal Tdap vaccination, with no cases of death reported to date in infants born to correctly vaccinated mothers (43). Dr. Amirthalingam also highlighted that any effects of maternal antibodies (MatAbs) in infant immune responses have not thus far translated into measurable impact on clinical disease in young children.

Sonali Kochhar (Global Healthcare Consulting, India) provided an overview of the study design and regulatory and safety considerations for maternal immunization clinical research in low- and middle-income countries. Beate Kampmann (Imperial College of London, United Kingdom) introduced the recently formed “Immunizing Pregnant Women and Infants” (IMPRINT) network, highlighting the purpose of the network, which brings together multidisciplinary stakeholders in the field to address specific scientific challenges (http://www.imprint-network.co.uk/).

### New and potential future targets for maternal and neonatal immunization.

**(i) Monoclonal antibodies.** James Crowe (Vanderbilt University, Nashville, TN, USA) suggested that in the next 5 years, Abs will be the new tools for the prevention of infectious diseases and rational, structure-based vaccine design will replace empirical antigen development. Better understanding of the shared elements of the human immune system is key to understanding the performance of vaccines and Abs. Although human genomes differ, he contends, Ab genes are much the same and the Abs we produce are very similar—thus, the study of one person`s responses can be generalized to others. Further, Ab technology is mature, rapid, and affordable, and there is experience of safety with Ab administration, which is conceptually much less complex than vaccines.

There are currently licensed prophylactic Abs for anthrax and RSV, and the pipeline includes next-generation RSV, Clostridium difficile, Pseudomonas aeruginosa, staphylococcal toxin, and influenza virus Abs. The first successful identification of therapeutic Abs was for Marburg virus, and there are ongoing studies for other hemorrhagic fevers and ZIKV. Potential new targets include pandemic influenza (for elderly and infants), seasonal influenza, ZIKV, GBS, pertussis, MRSA, nosocomial infections (e.g., *Acinetobacter* sp.), others currently listed as WHO agents of concern (e.g., Rift Valley fever virus, Lassa fever virus, Pan “flavivirus”), MERS CoV, and therapeutic rabies virus.

Structure-based vaccines are another option for developing new vaccines. New epitopes are the alternative way to identify new candidates instead of whole infectious agents. The Human Immunome Project (http://www.humanvaccinesproject.org/work/human-immunome-program/) aims to determine the sequence of every human Ab and T cell receptor. The human immunome comprises the complete set of recombined immune receptor (T and B cell receptor) genes and is much larger than the human genome. The goals of the Human Immunome Project are to define the number of sequences; stability of repertoires over time; proportion of shared immunome; effects of age, race/ethnicity, gender, and geography; and effects of vaccination and diseases on the immunome and to define its complete structure and functional catalogue. The project will allow investigators to better profile the response to vaccines, understand the genetic features underlying variability in response, facilitate rational structure-based design, and identify new Ab-based therapeutics.

**(ii) Vaccines for malaria and enteric pathogens.** Sophie Roetynk (MRCG at LSHTM, The Gambia) presented the promising results of a randomized controlled trial on the safety and immunogenicity of a malaria adenovirus-vectored vaccine (ChAd63/MVA ME-TRAP) in infants and neonates. The vaccine was safe and well tolerated, resulting in high-level immunogenicity, with the highest peak response observed in infants vaccinated at 8 weeks of age, and no interference with EPI vaccines (44). Esther Ndungo (University of Maryland School of Medicine, USA) showed the results of a study evaluating infant protection against shigellosis by naturally acquired and vaccine-induced maternal immunity in a mouse model. *Shigella* is a primary cause of diarrhea in children <5 years of age according to findings of the Global Enteric Multicenter Study (GEMS). In a mouse model of maternal antibody (MatAb) transfer and challenge study, Dr. Ndungo demonstrated that *Shigella* antigen-specific IgG and functional Abs can be transferred to pups via placenta and milk of immune mothers, that maternal vaccination (systemic IgG) protects infant mice against mucosal infection, and that protection persists beyond weaning. A seroepidemiology cohort study in maternal-infant pairs is being conducted in Malawi to evaluate the presence of invasion plasmid antigen B (IpaB)- and lipopolysaccharide (LPS)-specific Abs in mothers, their transplacental transfer to newborns, duration in infants, and correlation with protection.

**(iii) Prevention of multidrug resistance to antibiotics.** Ajoke Sobanjo-ter Meulen (BMGF, USA) emphasized that globally, the neonatal mortality rate is declining more slowly than the childhood mortality rate. She also noted growing numbers of facility-based births, especially in India, with significant implications for health care-associated neonatal infections. She showed data from the Delhi Neonatal Infection Study (DeNIS) collaboration from 3 hospitals in Delhi with high antimicrobial resistance rates among neonatal pathogens, especially Gram-negative bacteria (45). Countries with the highest mortality rates also demonstrate higher sepsis death rates as well as higher prematurity and intrapartum stillbirth rates. These outcomes may reflect underlying infection, and evidence to support this hypothesis exists from the CHAMPS study (https://champshealth.org). A recent review has now estimated the burden of global deaths due to sepsis with antimicrobial-resistant (AMR) organisms (46). Maternal vaccines (especially RSV and GBS) have the potential to reduce AMR by reducing the empirical use of antibiotics. Regarding GBS strains, she mentioned rising rates of resistance to second-line antibiotics such as macrolides and tetracycline. She noted the large number of women in high-income countries who are receiving antibiotics in labor in order to prevent (early-onset) GBS disease. Thus, effective vaccines could potentially reduce the numbers of infections, reduce the need for antibiotics, reduce the emergence of resistant strains, and improve prevention of resistant strains. Theoretically, a vaccine could be used to target determinants of antibiotic resistance, and vaccines against important Gram-negative pathogens are already on the horizon (47). The development of monoclonal antibodies (MAbs) at birth is also part of the Bill & Melinda Gates Foundation strategy, whereas prophylactic MAbs could be given to infants determined to be at risk. MAbs may reduce the need for or the duration of antibiotics. This approach could potentially be combined with maternal immunization against the pathogens discussed above.

**Panel discussions.** The meeting concluded with two panel discussions. The first panel focused on the topic “Implementing and Integrating Maternal and Infant Immunization: Challenges and Opportunities.” Participants were asked to address the following key questions:

- What are the key challenges for the implementation of maternal immunization programs worldwide?
- Is it feasible to, and what are the key challenges for integrating maternal and infant immunization schedules?
- What are critical knowledge gaps that need to be addressed in order to ensure a successful implementation of maternal immunization programs that complement infant immunization programs?
- Based on your expertise, what are potential solutions to the challenges and knowledge gaps you have identified?

The outcomes of this panel are described in Table 1.

**TABLE 1:**
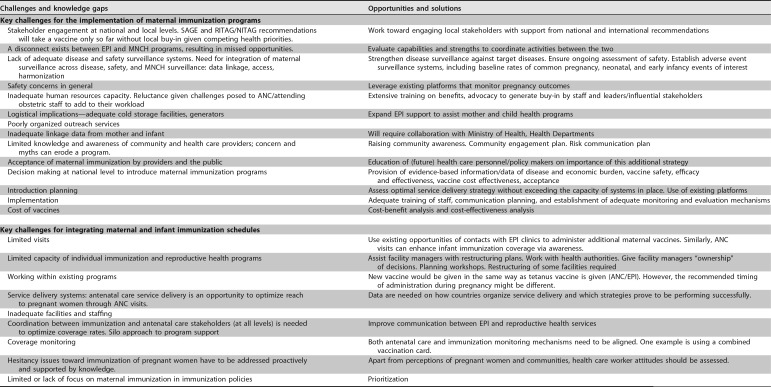

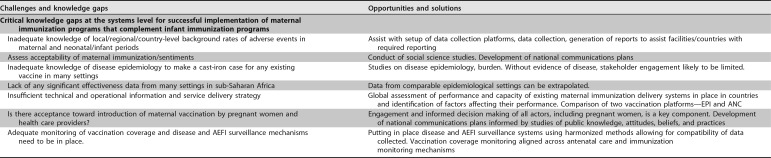
Challenges and opportunities to implement and integrate maternal and infant immunization^*a*^

a Participants included Azhar Abid Raza (UNICEF), Edwin Asturias (University of Colorado, Denver), Nele Berthels (FAMHP, Belgium), Ed Clarke (MRC Unit, The Gambia), Claire Cutland (University of the Witwatersrand), Linda Eckert (University of Washington, Seattle, WA, USA), Philipp Lambach (World Health Organization), and Elke Leuridan (University of Antwerp), representing leading public health organizations, regulatory, and obstetric and pediatric clinical investigators, with expertise in vaccinology, epidemiology, and public health. The session was chaired by Flor M. Munoz (Baylor College of Medicine, Houston, TX, USA). Abbreviations: SAGE, Strategic Advisory Group of Experts; RITAG, Regional Immunization Technical Advisory Group; NITAG, National Immunization Technical Advisory Group; EPI, Expanded Program on Immunization; MNCH, Maternal Newborn and Child Health; ANC, Antenatal Care; AEFI, Adverse Event Following Immunization; LMIC, low- and middle-income countries.

In addition, the panel was asked if they considered it feasible to integrate maternal and infant immunization schedules. The response was that in principle, this should be feasible in many countries, taking into consideration the success of the maternal-neonatal tetanus program. In order to achieve this, the panel suggested the following:

- Develop a maternal immunization investment case to depict the concept and advantages of maternal immunization, using maternal tetanus vaccination as an example (commonly appreciated in LMIC) and substantiated by sound country-specific evidence on disease burden.
- Identification of “champions” to push the maternal immunization agenda, especially regionally and at country level.
- Promotion of functional integration between EPI and Maternal Neonatal and Child Health (MNCH) programs using all available means, with national governments driving the process. Ideally equipping ANC clinics with immunization facilities—logistics and skills.
- A strong maternal immunization platform is required that normalizes vaccination practice among obstetric care providers and is supported by basic and continuing education, communication strategy, and a broad range of research.
- Resource requirements’ implications, including human resources and funding, should be adequately assessed and made available. Early and timely availability of resources for program implementation and community awareness will be instrumental.
- Involvement of professional associations, such as of obstetricians and gynecologists and public health physicians with ongoing sensitization.
- Advocacy and capacity building; manufacturers and health care providers to be explicit in providing safety information that specifically relates to product use in pregnancy or neonates

The second panel discussion focused on identifying “Key Messages from Stakeholders.” The panel members were chosen to represent the multiple disciplines relevant to this field. All panelists had prepared a statement summarizing 3 key challenges in their area, and proposed solutions, which are summarized in Table 2.

**TABLE 2:** Key messages from stakeholders^*a*^

Stakeholder	Main obstacles	Potential solutions
Investigators	Lack of baseline data for maternal and neonatal outcomes	Partnering with local care providers and the community, including women who have received vaccines in pregnancy as advocates for the program/study.
	Retention of pregnant women in clinical trials	Increase public acceptance and confidence through information.
	Impact of coinfections and adverse event reporting	Conduct multidisciplinary research which includes pregnant women and their communities.
Obstetric antenatal care providers	Acceptance and logistics of vaccination during pregnancy in LMIC	In some cases, the preferred time to give the vaccines is outside pregnancy, in adolescence, as part of a preconception package.
		Communication with the pregnant women needs to be “right the first time.”
		Harmonize standards for conduct of clinical trials and data collection systems so that not every country will wish to repeat the studies and results are acceptable across countries.
Regulatory perspective	Logistics of research and managing perceptions and expectations	The clinical development of novel vaccines requires frequent interactions with the Regulator, including DSMB and ethics input.
		Ethics committees need to be strengthened and need to include adequate multidisciplinary and regional representation of relevant experts. They may benefit from educational efforts on maternal immunization, especially on new data, experiences, and regulations from other maternal vaccine development programs.
		When a double-blind, placebo-controlled trial is not possible, do not refrain from doing studies altogether.
Academics	Support for research	Funding is needed for basic science but also for collection of baseline epidemiological data, which are hard to come by.
		Collaborations need to occur between all stakeholders.
		Postimplementation studies: effectiveness, coverage studies.
Policy	Challenges identified in different domains	
Biological issues	Timing of vaccination during pregnancy	Time of vaccination will determine titers of protective antibody at the time of delivery—encourage early registration to optimize the window.
	Impact on vaccination schedules	Impact of novel vaccines on any existing vaccines already in the schedule needs to be clarified, to inform scheduling and safety data.
Health systems issues	Logistics of antenatal care and EPI	Maternal vaccination is a golden opportunity to bring ANC and EPI together.
		The knowledge of front-line health care workers administering vaccines needs to improve through appropriate training.
		An investment case needs to be made, as policy relates to costs.
Sociocultural issues	Optimizing sharing of knowledge and implementation	Early pregnancy remains secretive in LMIC.
		Inform SAGE and prepare appropriate follow-up studies and have policy impact.
		Communication is key to educate on risk/benefit considerations and merits of vaccines. Uncertainties ought to be discussed, and risk/benefit considerations should be in the public domain through informed sources.
Industry	Collaborations	Industry needed to guarantee sustained supply of vaccines.
	Safety	Trust is required in provider, product, and policy.
		Licensing of products in pregnancy can remain a problem for industry.
		Safety monitoring of vaccines is continuous; safety data from special populations need to be enhanced (e.g., label updates).
Funders	Priorities and commitment	There is continued funder commitment, and there have been significant achievements.
		The maternal immunization platform has high potential for addressing neonatal deaths, preterm births, and stillbirths; all are the focus of funder activities.
		The maternal immunization platform is a potential means to combat antimicrobial resistance.
		Maternal immunization is a concept for cross-sectional learning between the vaccine and the maternal/neonatal health communities.
		Assay standardization commitments will inform label extensions.
		Maternal immunization fits well into the lifetime vaccination approach taken by PAHO and SAGE.
		The maternal immunization platform can serve a number of purposes toward improving maternal and infant health.
		Conducting basic science projects relating to maternal/fetal physiology/immunology.

a Participants included Mustapha Bittaye (ANC provider, MRC Unit, The Gambia), Sonali Kochhar (Researcher, Global Healthcare Consulting), Kirsty LeDoare (Academic Scientist, Imperial College London), Pieter Neels (Regulatory, Vaccine-Advice BVBA), Martin Ota (Policy Expert, WHO Africa Regional Office), Ajoke Sobanjo ter-Meulen (Funder, Bill and Melinda Gates Foundation), and Ivo Vojtek (Industry, GSK Vaccines). The session was chaired by Beate Kampmann (Researcher, Imperial College London and MRC Unit, The Gambia). Abbreviations: SAGE, Strategic Advisory Group of Experts; INMIS, International Neonatal and Maternal Immunization Symposium; LMIC, low- and middle-income countries; EPI, Expanded Program on Immunization; NIH, National Institutes of Health.

### Concluding remarks.

Basic science is evolving quickly, providing new insights on mechanisms of protection of mothers and young infants through maternal immunization. Clear challenges and goals were formulated by participants at the meeting, spanning basic sciences, clinical research, and implementation work; progress in all areas is needed to enhance our knowledge about vaccines currently recommended for maternal and neonatal immunization and neonatal immune development to derive rational future targets for vaccination during pregnancy and early life. The meeting’s deliberations actively contributed toward the goal of global implementation and integration of maternal and infant vaccination strategies.
